# Activation of 5-HT2B-receptors leads to increased vasodilation in mouse dura mater blood vessels

**DOI:** 10.1186/1129-2377-14-S1-P78

**Published:** 2013-02-21

**Authors:** D Segelcke, C Schulte, M Kremser, A Hunfeld, H Lübbert

**Affiliations:** 1 Ruhr University Bochum, LS Animal Physiology, Germany

## 

Neurogenic inflammation occurring in migraine may be associated with plasma protein extravasation (PPE) and vasodilation of dural blood vessels. 5-HT2B receptors may be involved in the onset of migraine attacks, in which receptor activation may induce increased synthesis of nitric oxide and, subsequently, the release of vasoactive CGRP from trigeminal nerve fibres. We found that the activation of 5-HT2B receptors in mice that were kept under hypoxic conditions for several weeks triggered an increased PPE in the dura mater. In addition to the PPE we now asked whether the activation of the 5-HT2B receptors also results in the vasodilation of dural blood vessels. For this purpose we combined intravital microscopy with the hypoxic mouse model. We removed a part of the skull in anesthetized mice and exposed the dura mater. Changes in blood vessel diameter have been observed via intravital microscopy after a topical application of substances directly onto the dura mater. Application of a 5-HT2B receptor agonist led to increased vasodilation of dural blood vessels. The effect could be completely blocked by specific receptor antagonists. The results confirm the involvement of 5-HT2B receptors in the onset of migraine attacks. While the topical application of CGRP alone was not sufficient to induce similar vasodilation in the mouse dura mater, the effect occurred when CGRP was applied after a preconstriction of the blood vessels by endothelin 1 (ET-1). Thus, we showed that the activation of 5-HT2B receptors not only induces PPE, but also dilates blood vessels in the dura mater. Whether and to which extent the vasodilation induced by 5-HT2B receptor activation is associated with the subsequent release of CGRP is subject to further studies.

